# Supplementary material for: Comparison of Retinal Structural and Neurovascular Changes between Patients with and without Amyloid Pathology
Source: J Clin Med. 2023 Feb 7;12(4):1310. doi: 10.3390/jcm12041310 (PMC9964845; doi:10.3390/jcm12041310)
Supplement: Supplementary file 1 [file jcm-12-01310-s001.zip › jcm-2180237-supplementary.pdf]

Supplementary Table S1. Demographics and clinical characteristics in each diagnostic groups according to Aβ pathology

	Dementia			MCI			CU		
	A+	A−	*P*-value	A+	A−	*P*-value	A+	A−	*P*-value
Number of eyes	22	5		17	18		3	6	
Age	73.68 ± 7.34	69.80 ± 10.55	0.344	68.59 ± 6.65	71.83 ± 6.30	0.184	74.67 ± 8.50	68.00 ± 5.73	0.262
Sex, female (%)	12 (54.5)	0 (0)	**0.047**	9 (52.9)	10 (55.6)	1.000	2 (66.7)	4 (66.7)	1.000
MMSE	19.05 ± 4.04	23.00 ± 4.30	0.055	25.18 ± 2.32	24.39 ± 3.24	0.590	25.33 ± 1.15	28.17 ± 1.47	**0.048**
CDR-SB	5.66 ± 2.54	4.30 ± 1.57	0.377	2.24 ± 1.15	1.86 ± 1.51	0.173	0.50 ± 0.50	0.42 ± 0.38	0.905
CDR	1.02 ± 0.36	0.80 ± 0.27	0.284	0.53 ± 0.12	0.53 ± 0.12	0.987	0.33 ± 0.29	0.33 ± 0.26	1.000
SBP, mmHg	129.09 ± 17.32	131.40 ± 12.36	0.976	131.29 ± 17.15	123.83 ± 13.31	0.153	153.00 ± 14.73	120.83 ± 16.68	**0.048**
DBP, mmHg	72.50 ± 12.03	76.20 ± 12.54	0.694	73.29 ± 11.55	70.07 ± 9.38	0.590	75.00 ± 8.89	71.00 ± 10.79	0.548
Hypertension (%)	9 (40.9)	2 (40.0)	1.000	7 (41.2)	6 (33.3)	0.733	1 (33.3)	0 (0)	0.333
Diabetes mellitus (%)	1 (4.5)	2 (40.0)	0.079	2 (11.8)	1 (5.6)	0.603	0 (0)	2 (33.3)	0.500
Dyslipidemia (%)	6 (27.3)	1 (20.0)	1.000	7 (41.2)	5 (27.8)	0.489	2 (66.7)	3 (50.0)	1.000
Eye laterality, right (%)	12 (54.5)	2 (40.0)	0.648	7 (41.2)	12 (66.7)	0.181	2 (66.7)	4 (66.7)	0.580
BCVA (logMAR)	0.15 ± 0.18	0.12 ± 0.13	0.896	0.14 ± 0.16	0.08 ± 0.09	0.598	0.00 ± 0.00	0.05 ± 0.08	0.289
SE, diopter	0.16 ± 1.24	–0.83 ± 3.32	0.876	0.23 ± 1.67	0.92 ± 1.64	0.364	–0.08 ± 0.44	0.75 ± 1.17	0.243
IOP, mmHg	13.50 ± 1.37	12.80 ± 1.30	0.456	14.53 ± 3.20	14.17 ± 2.71	0.688	14.67 ± 3.51	14.67 ± 3.45	1.000
Axial length, mm	23.52 ± 0.55	24.04 ± 0.68	0.182	23.38 ± 0.71	23.48 ± 0.89	0.575	23.20 ± 1.29	23.20 ± 0.90	1.000
CCT, µm	524.82 ± 27.38	542.20 ± 10.43	0.070	532.59 ± 30.62	521.94 ± 22.44	0.499	551.33 ± 23.80	528.17 ± 23.39	0.302

Values are presented as mean ± standard deviation or number (%) unless otherwise indicated. The significant differences between the two groups based on amyloid pathology were marked in bold. Aβ, beta amyloid; MCI, mild cognitive impairment; CU, cognitively unimpaired; A, beta amyloid; MMSE, Mini-Mental State Examination; CDR, Clinical Dementia Rating scale; CDR-SB, CDR-Sum of Boxes; SBP, systolic blood pressure; DBP, diastolic blood pressure; logMAR, logarithm of the minimum angle of resolution; SE, spherical equivalent; IOP, intraocular pressure; CCT, central corneal thickness.

Supplementary Table S2. Comparison of OCT and OCT angiography parameters in three groups

	Groups				
	Dementia	MCI	CU	*P*-value	**FDR_*P*-value**
Retinal structural factor					
Superior quadrant cpRNFLT, µm	110.41 ± 15.07[a]	115.09 ± 17.22	125.78 ± 12.67	0.019	0.249
5 clock-hour cpRNFLT, µm	87.48 ± 20.29[a]	92.83 ± 14.11	102.67 ± 14.47	0.032	0.249
6 clock-hour cpRNFLT, µm	116.33 ± 25.21[a]	128.83 ± 20.75	141.44 ± 21.10	0.019	0.249
Retinal vascular factor					
FAZ circularity index	0.63 ± 0.09[a]	0.65 ± 0.11[a]	0.74 ± 0.07	0.012	0.249
Perifoveal Temporal VD, mm/mm^2	14.56 ± 2.83[a]	15.10 ± 3.10	17.02 ± 2.32	0.046	0.249
Perifoveal Mean VD, mm/mm^2	16.20 ± 2.21[a]	16.67 ± 1.76	17.67 ± 1.94	0.040	0.249
Perifoveal Superior PD, %	0.40 ± 0.07[a]	0.41 ± 0.06	0.45 ± 0.05	0.048	0.249
Perifoveal Temporal PD, %	0.36 ± 0.07[a]	0.37 ± 0.08	0.42 ± 0.06	0.046	0.249
Perifoveal Mean PD, %	0.40 ± 0.06[a]	0.41 ± 0.04	0.44 ± 0.05	0.027	0.249

Values are presented as mean ± standard deviation. The significantly different parameters among the three clinical diagnostic groups were presented. The significant differences for post hoc analysis among the three clinical diagnostic groups indicated as follow: [a] $p < 0.0167$ versus CU; [b] $p < 0.0167$ versus MCI. cpRNFLT, circumpapillary retinal nerve fiber layer thickness; CU, cognitively unimpaired; FAZ, foveal avascular zone; MCI, mild cognitive impairment; FDR, false discovery rate; OCT/A, optical coherence tomography/angiography; PD, perfusion density; VD, vessel density; *P*, p-values not corrected for multiple testing; FDR_P, Benjamini-Hochberg corrected p-values for 52 OCT/OCTA variables

Supplementary Table S3. Binary logistic regression analyses adjusted for age and gender for predictors of dementia and/or MCI from CU, and for age, gender and MMSE for predictors of Aβ positive (A+) from Aβ negative (A−)

	Estimate	SE	Z	*P*-value	FDR_*P*-value
Dementia and MCI (n=62 eyes) vs CU (n=9 eyes)					
Superior quadrant cpRNFLT, μm	−0.064	0.031	−2.027	**0.043**	0.325
5 clock-hour cpRNFLT, μm	−0.035	0.022	−1.603	0.109	0.325
FAZ circularity index	−12.024	5.046	−2.383	**0.017**	0.292
Perifoveal Superior VD, mm/mm^2	−0.430	0.278	−1.546	0.122	0.346
Perifoveal Temporal VD, mm/mm^2	−0.381	0.200	−1.900	0.057	0.346
Perifoveal Inferior VD, mm/mm^2	−0.445	0.264	−1.689	0.091	0.346
Perifoveal Mean VD, mm/mm^2	−0.503	0.313	−1.606	0.108	0.346
Perifoveal Superior PD, %	−18.124	10.925	−1.659	0.097	0.224
Perifoveal Temporal PD, %	−14.158	7.691	−1.841	0.066	0.224
Perifoveal Inferior PD, %	−19.241	10.690	−1.800	0.072	0.224
Perifoveal Mean PD, %	−20.257	12.296	−1.647	0.099	0.224
Total mean VD, mm/mm^2	−0.486	0.307	−1.583	0.113	0.346
Total mean PD, %	−19.833	12.237	−1.621	0.105	0.224
Dementia (n=27 eyes) vs CU (n=9 eyes)					
Superior quadrant cpRNFLT, μm	−0.092	0.044	−2.091	**0.037**	0.207
5 clock-hour cpRNFLT, μm	−0.033	0.024	−1.358	0.174	0.424
6 clock-hour cpRNFLT, μm	−0.044	0.021	−2.108	**0.035**	0.207
FAZ circularity index	−21.782	8.431	−2.584	**0.010**	0.166
Perifoveal Temporal VD, mm/mm^2	−0.382	0.197	−1.940	0.052	0.397
Perifoveal Mean VD, mm/mm^2	−0.491	0.315	−1.557	0.119	0.397
Perifoveal Superior PD, %	−17.144	10.914	−1.571	0.116	0.264
Perifoveal Temporal PD, %	−14.326	7.627	−1.878	0.060	0.264
Perifoveal Mean PD, %	−18.722	11.999	−1.560	0.119	0.264
MCI (n=35 eyes) vs CU (n=9 eyes)					
FAZ circularity index	−10.255	5.057	−2.028	**0.043**	0.380
A+ (42 eyes) vs A− (29 eyes)					
Parafoveal Temporal VD, mm/mm^2	−0.252	0.122	−2.063	**0.039**	0.332
Parafoveal Inferior VD, mm/mm^2	−0.127	0.119	−1.069	0.285	0.441
Parafoveal Mean VD, mm/mm^2	−0.203	0.124	−1.633	0.102	0.353
Parafoveal Temporal PD, %	−11.306	4.798	−2.356	**0.018**	0.221
Parafoveal Inferior PD, %	−4.777	4.627	−1.032	0.302	0.402
Parafoveal Mean PD, %	−8.229	4.888	−1.684	0.092	0.369
Perifoveal Temporal VD, mm/mm^2	−0.210	0.102	−2.065	**0.039**	0.332
Perifoveal Temporal PD, %	−7.990	3.938	−2.029	**0.042**	0.255
Total mean PD, %	−6.881	5.380	−1.279	0.201	0.392

cpRNFLT, circumpapillary retinal nerve fiber layer thickness; CU, cognitively unimpaired; estimate, estimate for logistic regression models; FAZ, foveal avascular zone; FDR, false discovery rate; MCI, mild cognitive impairment; OCT/A, optical coherence tomography/angiography; PD, perfusion density; SE, standard error; VD, vessel density; Z = z-statistics of logistic regression model; *P*, p-values not corrected for multiple testing; FDR_P, Benjamini-Hochberg corrected p-values for 52 OCT/OCTA variables.

Supplementary Table S4. Comparison of OCT and OCTA parameters according to Aβ positivity

	Aβ positive (A+)	Aβ negative (A−)	*P*-value	*P*-value*	**FDR_*P*-value**
Entire group					
Parafoveal Temporal VD, mm/mm^2	15.95 ± 2.37	17.04 ± 2.06	0.029	**0.032**	0.218
Parafoveal Inferior VD, mm/mm^2	16.76 ± 1.97	17.33 ± 2.49	0.011	0.285	0.165
Parafoveal Mean VD, mm/mm^2	16.26 ± 2.03	17.05 ± 2.31	0.019	0.089	0.190
Parafoveal Temporal PD, %	0.38 ± 0.06	0.41 ± 0.06	0.009	**0.013**	0.165
Parafoveal Inferior PD, %	0.40 ± 0.05	0.41 ± 0.07	0.024	0.315	0.206
Parafoveal Mean PD, %	0.39 ± 0.05	0.41 ± 0.06	0.016	0.080	0.190
Perifoveal Temporal VD, mm/mm^2	14.43 ± 3.02	16.15 ± 2.63	0.007	**0.031**	0.165
Perifoveal Temporal PD, %	0.35 ± 0.08	0.40 ± 0.07	0.009	**0.034**	0.165
Total mean PD, %	0.39 ± 0.05	0.41 ± 0.05	0.045	0.185	0.300
MCI					
9 clock-hour cpRNFLT, µm	58.06 ± 7.95	51.67 ± 8.02	0.020	**0.029**	0.440
Temporal quadrant cpRNFLT, µm	70.71 ± 8.32	64.44 ± 11.45	0.045	0.074	0.450
Parafoveal Temporal VD, mm/mm^2	15.05 ± 2.91	16.97 ± 2.14	0.032	**0.043**	0.450
Parafoveal Temporal PD, %	0.35 ± 0.07	0.41 ± 0.06	0.014	**0.022**	0.440
Perifoveal Temporal VD, mm/mm^2	13.87 ± 3.56	16.25 ± 2.10	0.022	**0.022**	0.440
Perifoveal Temporal PD, %	0.34 ± 0.09	0.40 ± 0.05	0.041	**0.024**	0.450
CU					
Average mGC/IPLT, µm	74.67 ± 2.08	83.17 ± 8.01	0.048	0.200	0.576
Superotemporal mGC/IPLT, µm	72.33 ± 4.73	82.00 ± 7.56	0.024	0.305	0.480
Inferior mGC/IPLT, µm	73.67 ± 1.53	83.00 ± 7.51	0.024	0.128	0.480
Inferotemporal mGC/IPLT, µm	75.00 ± 2.65	84.67 ± 7.17	0.024	0.143	0.480
FAZ circularity index	0.66 ± 0.07	0.77 ± 0.04	0.048	0.255	0.576

Values are presented as mean ± standard deviation. *Adjusted for MMSE. Aβ, beta amyloid; cpRNFLT, circumpapillary retinal nerve fiber layer thickness; CU, cognitively unimpaired; FAZ, foveal avascular zone; FDR, false discovery rate; MCI, mild cognitive impairment; mGC/IPLT, macular ganglion cell/inner plexiform layer thickness; OCT/A, optical coherence tomography/angiography; PD, perfusion density; VD, vessel density; *P*, p-values not corrected for multiple testing; FDR_P, Benjamini-Hochberg corrected p-values for 52 OCT/OCTA variables.

Supplementary Table S5. Diagnostic performance of OCT and OCTA parameters according to Aβ pathology

	Cut-off	AUC	Sensitivity	Specificity
A+ (42 eyes) vs A− (29 eyes)				
Parafoveal Temporal VD, mm/mm^2	16.91	0.654	0.643	0.655
Parafoveal Inferior VD, mm/mm^2	17.98	0.678	0.857	0.552
Parafoveal Mean VD, mm/mm^2	17.25	0.665	0.691	0.724
Parafoveal Temporal PD, %	0.40	0.683	0.643	0.724
Parafoveal Inferior PD, %	0.43	0.658	0.738	0.621
Parafoveal Mean PD, %	0.42	0.668	0.762	0.655
Perifoveal Temporal VD, mm/mm^2	16.88	0.688	0.857	0.552
Perifoveal Temporal PD, %	0.41	0.684	0.857	0.586
Total mean PD, %	0.44	0.640	0.881	0.483
A+ MCI (17 eyes) vs A− MCI (18 eyes)				
9 clock-hour cpRNFLT, μm	54.00	0.727	0.706	0.778
Temporal quadrant cpRNFLT, μm	62.00	0.699	0.824	0.556
Parafoveal Temporal VD, mm/mm^2	16.88	0.712	0.765	0.722
Parafoveal Temporal PD, %	0.41	0.742	0.824	0.667
Perifoveal Temporal VD, mm/mm^2	16.88	0.725	0.882	0.500
Perifoveal Temporal PD, %	0.41	0.703	0.882	0.556
A+ CU (3 eyes) vs A− CU (6 eyes)				
Average mGC/IPLT, μm	77.00	0.944	1.000	0.833
Superotemporal mGC/IPLT, μm	76.00	0.972	1.000	0.833
Inferior mGC/IPLT, μm	75.00	0.972	1.000	0.833
Inferotemporal mGC/IPLT, μm	77.00	1.000	1.000	1.000
FAZ circularity index	0.71	0.944	1.000	0.833

A+, Aβpositive; A−, Aβ negative; AUC, area under the curve; cpRNFLT, circumpapillary retinal nerve fiber layer thickness; CU, cognitively unimpaired; FAZ, foveal avascular zone; MCI, mild cognitive impairment; mGC/IPLT, macular ganglion cell/inner plexiform layer thickness; OCT/A, optical coherence tomography/angiography; PD, perfusion density; VD, vessel density.

Supplementary Table S6. Demographics and clinical characteristics: A+ Dementia, A+ MCI, A− CU

	A+ Dementia	A+ MCI	A− CU	*P*-value
No. of eyes	22	17	6	
Age	73.68 ± 7.34	68.59 ± 6.65	68.00 ± 5.73	0.036
Sex, female (%)	12 (54.5)	9 (52.9)	4 (66.7)	0.837
MMSE	19.05 ± 4.04^{a,b}	25.18 ± 2.32	28.17 ± 1.47	**<0.001**
CDR-SB	5.66 ± 2.54^{a,b}	2.24 ± 1.15	0.42 ± 0.38	**<0.001**
CDR	1.02 ± 0.36^{a,b}	0.53 ± 0.12	0.33 ± 0.26	**<0.001**
SBP, mmHg	129.09 ± 17.32	131.29 ± 17.15	120.83 ± 16.68	0.378
DBP, mmHg	72.50 ± 12.03	73.29 ± 11.55	71.00 ± 10.79	0.954
Hypertension (%)	9 (40.9)	7 (41.2)	0 (0)	0.148
Diabetes mellitus (%)	1 (4.5)	2 (11.8)	2 (33.3)	0.138
Dyslipidemia (%)	6 (27.3)	7 (41.2)	3 (50.0)	0.487
Eye laterality, right (%)	12 (54.5)	7 (41.2)	4 (66.7)	0.508
BCVA (logMAR)	0.15 ± 0.18	0.14 ± 0.16	0.05 ± 0.08	0.420
SE, diopter	0.16 ± 1.24	0.23 ± 1.67	0.75 ± 1.17	0.543
IOP, mmHg	13.50 ± 1.37	14.53 ± 3.20	14.67 ± 3.45	0.550
Axial length, mm	23.52 ± 0.55	23.38 ± 0.71	23.20 ± 0.90	0.524
CCT, µm	524.82 ± 27.38	532.59 ± 30.62	528.17 ± 23.39	0.741

Values are presented as mean ± standard deviation or number (%) unless otherwise indicated. The significant differences for post hoc analysis among the three groups indicated as follow: ^{a} $p < 0.0167$ versus A− CU; ^{b} $p < 0.0167$ versus A+ MCI. A+, beta amyloid positive; A−, beta amyloid negative; BCVA, best corrected visual acuity; CCT, central corneal thickness; CDR, Clinical Dementia Rating scale; CDR-SB, CDR-Sum of Boxes; CU, cognitively unimpaired; DBP, diastolic blood pressure; IOP, intraocular pressure; logMAR, logarithm of the minimum angle of resolution; MCI, mild cognitive impairment; MMSE, Mini-Mental State Examination; SBP, systolic blood pressure; SE. spherical equivalent.

Supplementary Table S7. Comparison of OCT/OCTA parameters of A+ Dementia, or A+ MCI vs A− CU

	A+ Dementia	A+ MCI	A− CU	*P*-value	FDR_*P*-value
Retinal vascular factor					
FAZ circularity index	0.62 ± 0.08[a]	0.66 ± 0.11[a]	0.77 ± 0.04	0.003	0.180
Parafoveal Superior VD, mm/mm^2	16.86 ± 2.48	15.48 ± 3.04[a]	17.99 ± 2.61	0.043	0.218
Parafoveal Temporal VD, mm/mm^2	16.57 ± 1.68	15.05 ± 2.91[a]	17.92 ± 1.12	0.017	0.218
Parafoveal Inferior VD, mm/mm^2	16.58 ± 2.20[a]	17.04 ± 1.72	18.39 ± 0.91	0.038	0.218
Parafoveal Temporal PD, %	0.39 ± 0.05	0.35 ± 0.07[a]	0.43 ± 0.03	0.008	0.218
Perifoveal Temporal VD, mm/mm^2	14.66 ± 2.67[a]	13.87 ± 3.56[a]	17.55 ± 2.40	0.022	0.218
Perifoveal Temporal PD, %	0.36 ± 0.07[a]	0.34 ± 0.09[a]	0.43 ± 0.06	0.024	0.218
Perifoveal mean PD, %	0.40 ± 0.06[a]	0.41 ± 0.04	0.44 ± 0.06	0.031	0.218
Total mean PD, %	0.39 ± 0.06[a]	0.39 ± 0.04	0.43 ± 0.06	0.046	0.218

Values are presented as mean ± standard deviation or number (%) unless otherwise indicated. The significantly different parameters among the three groups were presented. The significant differences for post hoc analysis among the three clinical diagnostic groups indicated as follow: [a] $p < 0.0167$ versus A− CU; [b] $p < 0.0167$ versus A+ MCI. A+, beta amyloid positive; A−, beta amyloid negative; CU, cognitively unimpaired; FAZ, foveal avascular zone; FDR, false discovery rate; MCI, mild cognitive impairment; mGC/IPLT, macular ganglion cell/inner plexiform layer thickness; OCT/A, optical coherence tomography/angiography; PD, perfusion density; VD, vessel density; *P*, p-values not corrected for multiple testing; FDR_P, Benjamini-Hochberg corrected p-values for 52 OCT/OCTA variables.
